# Integrin β6 expression in colorectal cancer cells promotes liver metastasis through enhanced adhesion to endothelial fibronectin

**DOI:** 10.1002/ijc.35504

**Published:** 2025-06-09

**Authors:** Chiara Van Passen, Julia Krug, Luisa Weiß, Mariam Mohamed Abdou, Philipp Tripal, Benjamin Schmid, René Krüger, Yanmin Lyu, Bisan Abdalfatah Zohud, Katja Petter, Carol Geppert, Susanne Merkel, Barbara Bärthlein, Philipp Busenhart, Michael Scharl, Elisabeth Naschberger, Michael Stürzl

**Affiliations:** ^1^ Division of Molecular and Experimental Surgery, Uniklinikum Erlangen Friedrich‐Alexander‐Universität (FAU) Erlangen‐Nürnberg Erlangen Germany; ^2^ Optical Imaging Competence Centre Erlangen (OICE) Friedrich‐Alexander‐Universität (FAU) Erlangen‐Nürnberg Erlangen Germany; ^3^ Department of Medicine 4 – Nephrology and Hypertension Uniklinikum Erlangen, Friedrich‐Alexander‐Universität (FAU) Erlangen‐Nürnberg Erlangen Germany; ^4^ Institute of Pathology Uniklinikum Erlangen, Friedrich‐Alexander‐Universität (FAU) Erlangen‐Nürnberg Erlangen Germany; ^5^ CCC WERA: Comprehensive Cancer Center Alliance WERA (CCC WERA) Erlangen Germany; ^6^ Departement of Surgery Uniklinikum Erlangen, Friedrich‐Alexander‐Universität (FAU) Erlangen‐Nürnberg Erlangen Germany; ^7^ Medical Centre for Information and Communication Technology Uniklinikum Erlangen Erlangen Germany; ^8^ Department of Gastroenterology and Hepatology University Hospital Zürich, University of Zürich Zürich Switzerland; ^9^ CCC Erlangen‐EMN: Comprehensive Cancer Center Erlangen‐EMN (CCC ER‐EMN) Erlangen Germany; ^10^ BZKF: Bavarian Cancer Research Center (BZKF) Germany; ^11^ Department of Dermatology, Venerology and Allergology University Hospital Würzburg Würzburg Germany

**Keywords:** adhesion, blood vessels, colorectal cancer, integrin β6, metastasis

## Abstract

Integrin β6 is associated with poor prognosis in colorectal cancer (CRC) patients, with metastasis being a crucial determinant. Capillary endothelial cells (EC) in the liver and lung are the primary sites of contact for circulating tumour cells during metastasis. Here, we analysed the role of integrin β6 in tumour cells for their interaction with EC. Integrin β6 functions as a heterodimer with integrin αv. Interestingly, we found that liver and lung EC strongly express fibronectin, a high‐affinity ligand of αvβ6. Expression of *ITGB6* in CRC tumour cells closely correlated with their adhesion to EC. This interaction was greatly reduced by silencing *ITGB6* in the tumour cells and was integrin β6 dependent under both static and flow conditions. Binding assays with fibronectin‐coated surfaces, competing RGD peptides, and integrin β6‐neutralizing antibodies confirmed the crucial role of β6‐fibronectin binding in the interaction between tumour cells and EC. Since metastatic tumours exhibit increased proteolytic activity, we examined integrin β6 stability under these conditions. Remarkably, β6 remained resistant to trypsin and the matrix metalloprotease 12, underscoring its role in maintaining tumour cell adhesion in proteolytic microenvironments. Furthermore, ITGB6 expression was significantly elevated in liver metastases compared to corresponding primary tumours from the same patients, suggesting an enrichment of β6‐expressing cells in metastatic sites. These results suggest that tumour cell integrin β6 binding to EC‐derived fibronectin may serve as a critical first step in metastasis formation. Targeting this interaction could provide a promising therapeutic strategy to repress CRC metastasis.

## What's New?

Integrins are critical mediators of cell adhesion and migration and have been implicated in cancer progression. Here, the authors show that endothelial cells in target organs of colorectal cancer metastasis express fibronectin, and that colorectal cancer tumour cells can bind fibronectin through integrin β6. This interaction is resistant to proteolytic microenvironments, is required for circulating tumour cell adhesion to blood vessels, and can be efficiently blocked using peptides and antibodies. Additionally, ITGB6‐expressing tumour cells are enriched in colorectal cancer liver metastases. Targeting integrin β6 could be a promising therapeutic strategy to repress metastasis in colorectal cancer patients.

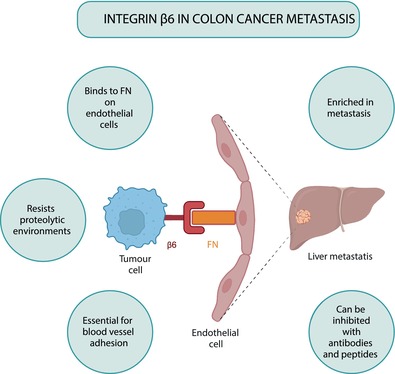

**Abbreviations**
BSA‐PBS: bovine serum albumin‐phosphate‐buffered saline
CRC: colorectal carcinoma
DNS: donkey normal serum
EC: endothelial cell
HUVEC: human umbilical vein endothelial cell
IHC: immunohistochemistry
KO: knockout
MMPs: matrix metalloproteases
qRT–PCR: quantitative reverse transcriptase PCR
RNS: rabbit normal serum

## 1 INTRODUCTION

Colorectal cancer (CRC) is the third most commonly diagnosed and fourth deadliest cancer worldwide.^1^ One in four patients with CRC has metastases at the time of diagnosis, and one in two patients will develop metastases during the course of the disease.^2^ Despite surgical and technological advances, the long‐term survival and cure rates of patients with metastatic CRC remain poor.^3^ There is an urgent need to understand the steps required for the establishment of CRC metastasis and the molecules involved to facilitate the development of novel therapeutic strategies. The metastatic spread of CRC comprises several steps by which cancer cells disseminate from the colon to a secondary growth site, most commonly the lung or liver.^4^ Tumour cells invade the stroma to reach the bloodstream, where, after anchorage‐independent survival, cell–cell interactions between the circulating tumour cells and the endothelial cells (EC) trigger the adhesion of tumour cells at the luminal side of the blood vessel endothelium in a distant organ, allowing extravasation, the formation of micrometastases and, ultimately, the formation of macrometastases.

Integrin β6 is involved in CRC pathogenesis.^5^, ^6^ It is not expressed under normal conditions in adults but is considerably upregulated during tumourigenesis, particularly in epithelial cancers, where its expression is directly associated with tumour progression.^7^ Integrin β6 can be detected in the serum, but it is unclear whether the whole protein or only sub‐fragments are present. Interestingly, high β6 serum concentrations indicate the presence of metastatic disease and poor prognosis in CRC patients,^8^ suggesting its potential use as a marker for the diagnosis, prognosis, and surveillance of CRC. Furthermore, integrin β6 is involved in immune evasion in CRC, causing inhibition of the antitumour immune response and resistance to immune checkpoint blockade therapy by activating latent transforming growth factor‐β (TGF‐β).^9^

Integrin β6 forms a heterodimer exclusively with integrin αv, forming the integrin αvβ6 receptor complex. In addition, integrin αv can interact with other integrins, such as integrin β1, integrin β3, integrin β5, and integrin β8.^10^

Integrin β6 is active in the metastatic cascade and contributes to aggressive disease. For example, integrin β6 enhances the migration^11^, ^12^ and invasion^13^, ^14^, ^15^, ^16^, ^17^ of tumour cells. Through TGF‐β activation, it can induce the epithelial‐to‐mesenchymal transition of tumour cells.^7^ Here, we propose that integrin β6 also plays a pivotal role in the adhesion of tumour cells to the vascular endothelium, potentially in the liver and lung, the primary target organs of metastasis. Integrins are a large family of transmembrane cell adhesion proteins that connect the extracellular matrix and the cytoskeleton or serve as cell‐to‐cell adhesion molecules. Accordingly, we propose that integrin β6 may promote the adhesion of disseminated tumour cells to EC lining blood vessels at metastatic sites. This process could be key for extravasation and metastatic colonization and may provide a new perspective for the suppression of metastasis in CRC patients.

## 2 MATERIALS AND METHODS

### 2.1 Patients

A total of 464 patients with Union for International Cancer Control (UICC) 2017 stage I–IV CRC were recruited prospectively between 2009 and 2012. Patients with preoperative radiation or chemotherapy, hereditary CRC (familial adenomatous polyposis, hereditary nonpolyposis colorectal cancer) or inflammatory bowel disease (Crohn's disease, ulcerative colitis) were excluded. The patients' characteristics are given in Tables S1–S4.

### 2.2 Cell culture

The DLD‐1 (RRID: CVCL_0248, male), HeLa (RRID: CVCL_0030, female), HT‐29 (RRID: CVCL_0320, female), RKO (RRID: CVCL_0504) and SW948 (RRID: CVCL_0632, female) cell lines were purchased from ATCC (Manassas, VA, USA). LOVO (RRID: CVCL_0399, male) cells were obtained from the DSMZ (Braunschweig‐Süd, Germany). The BL‐70 cells (RRID: CVCL_1088, male) were a gift from Prof. Dr. A. Kieser (Research Unit Signaling and Translation, Helmholtz Center Munich ‐ German Research Center for Environmental Health, Neuherberg, Germany) and PC‐3 (RRID: CVCL_0035, male) was gifted by Prof. Dr. H. Taubert (Department of Urology and Pediatric Urology, University Hospital Erlangen, Friedrich‐Alexander Universität Erlangen‐Nürnberg, Erlangen, Germany). Human umbilical vein endothelial cells (HUVEC, RRID: CVCL_2959, pooled patients) were obtained from PromoCell (Heidelberg, Germany) and primary human liver (HLECP1) and lung microvascular (CC‐2527) EC were obtained from Lonza (Basel, Switzerland).

The cells were cultured according to the manufacturer's instructions as follows: BL‐70 (RPMI 1640, 10% FCS, 5% CO_2_); DLD‐1 (RPMI 1640, 10% FCS, 5% CO_2_); HeLa (DMEM, 10% FCS, 8.5% CO_2_); HT‐29 (McCoy, 10% FCS, 5% CO_2_); LOVO (RPMI 1640, 10% FCS, 5% CO_2_); PC‐3 [Ham's F‐12 K (Kaighn's) Medium, 10% FCS, 5% CO_2_]; RKO (MEM, 10% FCS, 5% CO_2_); SW948 (L‐15, 10% FCS, 0% CO_2_) (all Thermo Fisher Scientific, Waltham, MA, USA); HUVEC (ECGM, C‐22010, PromoCell, 2% FCS, 5% CO_2_); liver EC (EGM™‐2, CC‐3162, Lonza, 2% FCS, 5% CO_2_); and lung EC (EGM™‐2 MV, CC‐3202, Lonza, 2% FCS, 5% CO_2_). All cell lines were authenticated using short tandem repeat (STR) profiling within the last 3 years.^18^ All experiments were performed with mycoplasma‐free cells.

### 2.3 CRISPR‐Cas9 gene editing

CRISPR‐Cas9 knockout of *ITGB6* expression in HT‐29 cells was performed according to previously published procedures.^19^ Single‐guide RNAs targeting the *ITGB6* gene and nontargeting controls were designed to generate knockout (KO) and control (non‐targeting; NT) cells. The sequence for the *ITGB6* single guide RNA is (5′ – 3′) CACACTGAGGTCCAATAAGC. Clones were screened for knockout of integrin β6 via western blotting.

### 2.4 Tumour cell adhesion

#### 2.4.1 Static adhesion assay

**FIGURE 1:**
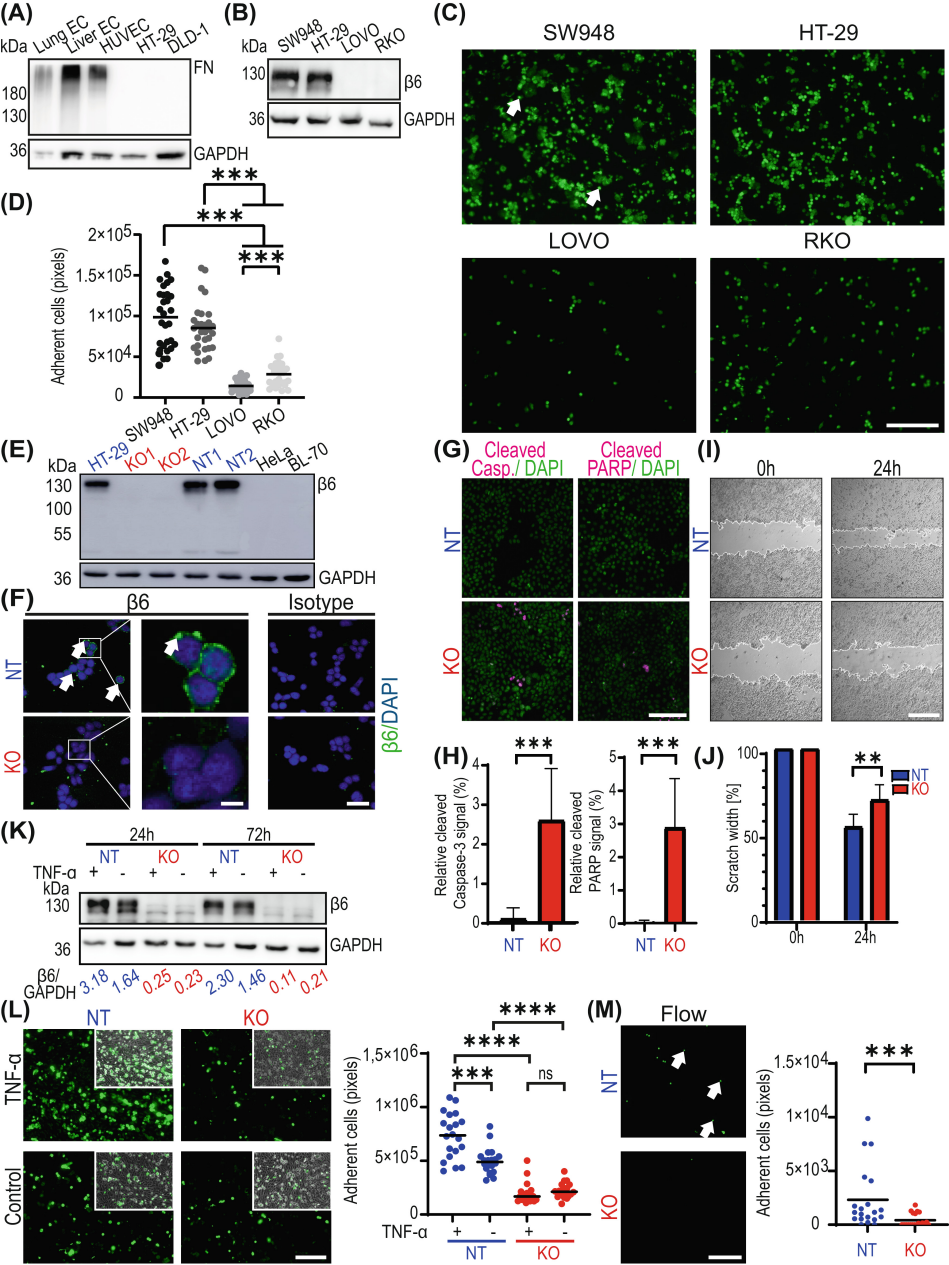
*ITGB6* expression in different CRC cell lines and *ITGB6* knockout cells is related to the adhesion of tumour cells to endothelial cells under static and flow conditions. (A) Lysates of lung EC, liver EC, HUVEC, HT‐29 and DLD1 cells were collected and analysed via western blotting with an anti‐fibronectin antibody. GAPDH was used as a loading control. (B) Lysates of CRC cells were collected and subjected to western blot analysis for β6 expression. GAPDH was used as a loading control. (C) CRC cell adhesion (arrows) to HUVEC under static conditions. Scale bar: 200 μm. (D) Quantitative evaluation of CRC cell adhesion to an HUVEC monolayer. Mann–Whitney *U* test; ***p < 0.001. (E) Lysates of HT‐29 cells with *ITGB6* KO and control cells (NT) were collected, and *ITGB6* expression was analysed via western blotting. HeLa and BL‐70 cells were used as a negative control for *ITGB6* expression. GAPDH was used as a loading control. (F) Cell surface staining for integrin β6 (green; arrows) and DAPI (blue). Scale bar: 50 μm; inset, 10 μm. (G) Apoptosis was induced with Nutlin‐3, and 24 h later, cells were stained using cleaved caspase‐3 (pink) and cleaved PARP (pink) antibodies and DAPI (green). Scale bar: 125 μm. (H) The cleaved caspase‐3 and cleaved PARP signals relative to the DAPI signals observed in G are reported as percentages. Mann–Whitney *U* test; ***p < 0.001. (I) A scratch was made in a confluent layer of HT‐29 cells. Cells were stimulated with hepatocyte growth factor (HGF), and the wound was allowed to close for 24 h. Scale bar: 500 μm. (J) Quantitative evaluation of the results shown in I. Unpaired *t*‐test; **p < 0.01. (K) Lysates of HT‐29 cells stimulated for 24 or 72 h with 50 ng/μL TNF‐α were collected and analysed via western blotting for β6 expression. The results of the quantification of the integrin β6 protein signals relative to those of GAPDH are given in the numbers below. (L) The capacity of TNF‐α‐treated HT‐29 KO and NT cells to adhere to an HUVEC monolayer after 2 h of incubation. Integrity of the HUVEC monolayer was controlled by phase contrast microscopy (insets). Scale bar: 250 μm. Mann–Whitney *U* test; ns, not significant; ***p < 0.001; ****p < 0.0001. (M) Representation and quantification of the capacity of *ITGB6* KO and NT cells to adhere to an HUVEC monolayer under flow conditions. Arrows indicate the adherent cells. Scale bar: 250 μm. Mann–Whitney *U* test; ***p < 0.001. In C, D, L, and M, IL‐1β‐stimulated HUVECs were used. In C, L, and M, CRC cells were stained using Cytopainter.

For the adhesion assays with EC, HUVEC (passage <7) were seeded into gelatine‐coated containments [1.7 × 10^5^ cells/well in 24‐well plates (142475, Thermo Fisher Scientific, Figures 1C,D,L and 3A–C); 1.5 × 10^5^ cells/chamber in chamber slides (354104, Falcon, Glendale, AZ, USA, Figure 5B)]. After 4 days, the HUVEC were stimulated with 100 ng/mL IL‐1β (H6291, Merck, Rahway, NJ, USA) for 24 h. In parallel, HT‐29 cells were seeded so that they reached 90% confluency by day 5. Afterwards, the tumour cells (1.6 × 10^5^) were harvested with 5 mM EDTA or, where indicated, with 0.05% trypsin in phosphate‐buffered saline (PBS), stained (cytopainter ab176735, Abcam, Cambridge, UK) and added to the HUVEC. After 2 h, nonadherent cells were removed by washing once with PBS. The cells were fixed with 4% PFA for 10 min and washed with PBS.

Where indicated, HT‐29 tumour cells were starved for 10 h and stimulated with 50 ng/mL TNF‐α (11371843001, Roche, Basel, Switzerland) for 6 h prior to addition to EC. For blocking experiments, the tumour cells were preincubated for 1 h at 37°C with the following reagents: mouse IgG2a isotype control (MAB003, R&D Systems, RRID: AB_357345, 10 μg/mL), mouse anti‐human ITGAVB6 antibody (MAB2077Z, Merck, RRID: AB_94538, 10 μg/mL) or RGD peptide (7723, R&D Systems, 100 μg/mL unless indicated otherwise).

For binding assays in the absence of EC, the wells of a 96‐well plate (Cat167425, Thermo Fisher Scientific) were coated overnight at 4°C with human fibronectin (Cat1918‐FN, R&D Systems, Minnesota, MPLS, USA, 30 μg/mL), human vitronectin (Cat2349‐VN, R&D Systems, 30 μg/mL) or human tenascin C (Cat3358‐TC, R&D Systems, 30 μg/mL) with an additional 0.1 μg/mL fibronectin only for the tenascin C condition. The next day, the plate was incubated at 37°C for at least 1 h. Then, the wells were washed with PBS, and nonspecific binding sites were blocked with 1% bovine serum albumin (BSA)‐PBS for 1 h at 37°C. Subsequently, 2 × 10^5^ tumour cells/mL were added, and further proceeding was carried out as above.

#### 2.4.2 Dynamic adhesion assay

The dynamic adhesion assay followed the protocol of the static adhesion assay but used channel slides [μ‐Slide I Luer (80186, Ibidi, Gräfelfing, Germany), Figure 1M] instead. The μ‐Slides with EC were connected to the perfusion pump (10902, Ibidi) with 13 mL of full endothelial cell growth medium (ECGM, C‐22010, PromoCell), and flow was applied (6.0 mbar pressure, 2.03 dyn/cm^2^ shear stress) 2 h after the cells were seeded. On day 5, the medium in the reservoirs was removed and replaced with 6 mL of a suspension containing 3.0 × 10^5^ cytopainter‐stained HT‐29 tumour cells per mL in each reservoir, and the flow was restarted.

In all cases, adherent cells were imaged using an epifluorescence microscope (DM6000 B, Leica, Wetzlar, Germany) and automatically quantified using a macro (https://github.com/ChiaraVP/Macro-for-quantifying-adherent-cells.git).

The use of different containments (24‐well plates, chamber slides, and channel slides) did not affect the differential adhesion of KO and NT cells to HUVEC.

### 2.5 Proteolytic digestion of recombinant proteins by matrix metalloproteases

MMP‐12 (R&D Systems, Catalogue #: 917‐MPB) was activated in a concentration of 50 ng/μL by incubation in TCN buffer (10 mM Tris, 150 mM NaCl, 5 mM CaCl2, pH 7.5) at 37°C for 2 h. Following enzyme activation, the recombinant human integrins (integrin αvβ6; Catalogue #: T6‐H52E1, integrin αvβ1; Catalogue #: IT1‐H52E1, integrin αvβ5; Catalogue #: IT5‐H52W5, AcroBiosystems, Newark, DE, USA) were added in an enzyme‐to‐substrate ratio of 1:10 in TCN buffer for 1 h (MMP‐12) at 37°C. Proteolytic digestion was halted by adding 4× Laemmli buffer containing 2‐mercaptoethanol, followed by a 5‐min heat denaturation at 95°C. The digested products were analyzed by Western blot.

### 2.6 Western blot analysis

Tumour cells were harvested at 90% confluence without starvation. For induction experiments (Figure 1K), tumour cells were starved for 6 h and stimulated with 50 ng/mL TNF‐α (11371843001, Roche) for 24 or 72 h. Protein extraction and quantification, western blotting, signal detection, and intensity determination were performed as described previously.^19^ The following antibodies were used for detection: rabbit anti‐human integrin αv (ab179475, Abcam, RRID: AB_2716738, 1:2000), rabbit anti‐human integrin β1 (12594‐1‐AP, Proteintech, Rosemont, IL, USA, RRID: AB_2130085, 1:5000), rabbit anti‐human integrin β5 (4708S, Cell Signaling, Danvers, MA, USA, RRID: AB_2129156, 1:1000), sheep anti‐human integrin β6 (AF4155, R&D Systems, RRID: AB_2129295, 1:2000), and rabbit anti‐human fibronectin (F3648, Merck, RRID: AB_476976, 1:1000). GAPDH (mouse anti‐human antibody, MAB374, Merck, RRID: AB_2107445, 1:40,000) was used as a loading control. A donkey anti‐sheep IgG‐HRP antibody (HAF016, R&D Systems, RRID: AB_562591) was used as the secondary antibody at a dilution of 1:2000, and a goat anti‐rabbit‐HRP antibody (P0448, Agilent, Santa Clara, CA, USA, RRID: AB_2617138) and a rabbit anti‐mouse‐HRP antibody (P0260, Agilent, RRID: AB_2636929) were used at a dilution of 1:5000. The blots were re‐probed after treatment with Restore™ Western Blot Stripping Buffer (21059, Thermo Fisher Scientific, 15 min incubation) to detect the loading control.

### 2.7 Immunoprecipitation

Proteins were harvested by scraping the cells in ice‐cold immunoprecipitation lysis buffer (20 mM Tris–HCl, pH 7.5, 150 mM NaCl, 5 mM MgCl_2_, 1% IGEPAL; supplemented with 1 tablet of Complete Mini EDTA‐free protease inhibitor cocktail [4693159001, Roche] per 10 mL), and protein concentrations were determined using a DC assay (Bio‐Rad Laboratories, München, Germany). One milligram of lysate was precleared via incubation with 20 μL of protein G Sepharose 4 Fast Flow (GE Healthcare, Chicago, IL, USA). For immunoprecipitation, 20 μL of protein G Sepharose 4 Fast Flow was preincubated with 1 μg of goat anti‐human ITGAV (AF1219, R&D Systems, RRID: AB_663829) for 30 min at 4°C and subsequently incubated with precleared lysates overnight on an overhead rotator at 4°C. The next day, the beads were washed five times for 10 min with ice‐cold immunoprecipitation wash buffer on an overhead rotator. After the final washing step, the beads were centrifuged, resuspended in 2× Laemmli, and boiled for 5 min at 100°C. The immunoprecipitated lysates were then subjected to western blot analysis.

### 2.8 Wound healing assay

HT‐29 cells were seeded at a density of 4.0 × 10^5^ cells/well into a 12‐well plate (150628, Thermo Fisher Scientific) in triplicate. Wounds were created by scratching the confluent cell layer in a straight line using a 100–1000 μL sterile pipette tip. McCoy's medium supplemented with 0.5% FBS was used to remove cell debris, and the cells were stimulated with hepatocyte growth factor (HGF) (11343413, ImmunoTools, Friesoythe, Germany, 40 ng/mL) for 24 h. Scratch closure was photographed after 0 and 24 h, and the scratch width was measured using ImageJ.^20^ Wound closure is given as the relative migration rate, which was calculated as the quotient of the wound width at the respective time point compared to 0 h.

### 2.9 Cleaved Caspase‐3 and PARP staining

HT‐29 cells were seeded at a density of 5.0 × 10^4^ cells/chamber into a 4‐well chamber slide (354104, Falcon). After 24 h, the cells were treated with 25 μM Nutlin‐3 (N6287, Sigma–Aldrich, St. Louis, MO, USA) to induce apoptosis. After 24 h, the cells were fixed with 10% PFA, permeabilized with 0.1% saponin for 30 min, and blocked with 10% GNS for 10 min. Immunostaining was performed via incubation with primary antibodies against cleaved rabbit anti‐human PARP (5625, Cell Signaling, RRID: AB_10699459, 1:400) and rabbit anti‐human cleaved caspase‐3 (9664, Cell Signaling, RRID: AB_2070042, 1:400) for 2 h, followed by incubation with an anti‐rabbit Alexa546 secondary antibody (A10040, Invitrogen, Waltham, MA, USA, RRID: AB_2534016, 1:500) for 45 min. Nuclei were stained with DAPI (D21490, Invitrogen, 1:5000) and DRAQ5 (4084, Cell Signaling, 1:1000), and slides were mounted with fluorescent mounting medium (S3023, Agilent). Images were acquired using a Leica SPE microscope, and ImageJ software was used for analysis. Area measurements of stained regions were recorded and analyzed to quantify apoptosis.

### 2.10 Immunohistochemical staining

For human IHC staining, archived formalin‐fixed, paraffin‐embedded human colorectal carcinoma tissues were cut into 4‐μm sections using a rotary microtome (Zeiss HM 355S, Zeiss, Oberkochen, Germany). The sections were deparaffinized at 60°C for at least 2 h and rehydrated via a graded series of ethanol (100% to 70%). The rehydrated samples were heated in antigen retrieval solution (S2369, Agilent, Target Retrieval Solution, pH: 6.0) in a water bath for 30 min at 95°C. Endogenous peroxidases were blocked with 7.5% H_2_O_2_ in demineralized water for 10 min, and endogenous biotin was blocked with an avidin/biotin blocking kit (SP‐2001, Biozol, Eching, Germany). Unspecific binding sites were blocked with 2.5% rabbit normal serum (RNS, 011‐000‐120, Jackson ImmunoResearch, West Grove, PA, USA). The sections were subsequently incubated with a 1:500 dilution of primary sheep anti‐human integrin β6 antibody (AF4155, R&D Systems, RRID: AB_2129295) or normal sheep anti‐human IgG control (500‐1‐A, R&D Systems, RRID: AB_10141430) in 0.5% RNS overnight at 4°C. Then sections were incubated with rabbit anti‐sheep biotinylated antibody (31840, Invitrogen, RRID: AB_228455) for 30 min at room temperature, followed by incubation with ABC reagent (Anti‐Rabbit IgG Vectastain Elite ABC‐Kit, PK‐6101, Biozol, RRID: AB_2336820) for 30 min at room temperature. Immunodetection was performed with a NovaRed substrate kit (SK‐4800, Vector Laboratories, Newark, CA, USA) for 10 min at room temperature. The sections were counterstained with haematoxylin (1.05174.1000, Sigma–Aldrich), dehydrated and mounted in Vectamount Permanent Mounting Medium (H‐5000, Vector Laboratories). The processed samples were examined via a microscope slide scanner (Nanozoomer S60, Hamamatsu Photonics, Hamamatsu City, Japan), and images were obtained at 20× magnification.

### 2.11 Immunofluorescence Staining

#### 2.11.1 Cell surface staining of integrin β6

The cells were seeded a minimum of 48 h before the staining was started. After the initial washes with PBS supplemented with Ca^2+^/Mg^2+^ and blocking with 10% donkey normal serum (DNS) in PBS supplemented with Ca^2+^/Mg^2+^, the primary antibody (anti‐human integrin β6, AF4155, R&D Systems, RRID: AB_2129295, 1:40) or isotype control antibody [sheep IgG (5‐001‐A, RRID: AB_10141430)] was applied for 2 h at 37°C. The secondary antibody, Alexa Fluor 488‐conjugated donkey anti‐sheep IgG (A‐11015, 1:500, RRID: AB_141362), was applied for 45 min at RT. The cells were washed, fixed for 15 min with 4% paraformaldehyde, counterstained with DRAQ5, and mounted with fluorescent mounting medium (S3023, Agilent). Immunofluorescent images were captured using a Leica SPE microscope.

#### 2.11.2 Staining of formaldehyde‐fixed β6 and fibronectin

HUVEC and adherent *ITGB6*‐expressing HT‐29 cells were fixed in buffered‐formaldehyde solution and blocked with 10% DNS in PBS for 10 min at 37°C. The primary antibodies, sheep anti‐human integrin β6 (AF4155, R&D Systems, RRID: AB_2129295, 1:40) and rabbit anti‐human fibronectin (F3648, Merck, RRID: AB_476976, 1:500) as well as the respective isotype control antibodies [sheep IgG (5‐001‐A, RRID: AB_10141430), rabbit IgG (AB172730, RRID: AB_2687931)], were applied for 2 h at 37°C. The secondary antibodies, Alexa Fluor 488‐conjugated donkey anti‐sheep (A‐11015, 1:500, RRID: AB_141362) and Alexa Fluor 546‐conjugated donkey anti‐rabbit (A‐10040, 1:500, RRID: AB_2534016) were added for 45 min at room temperature (RT). Cells were counterstained with DRAQ5 and mounted using fluorescent mounting medium (S3023, Agilent). Immunofluorescent images were captured using a Leica SP5 laser scanning microscope.

### 2.12 RNA isolation and quantitative reverse‐transcription polymerase chain reaction

RNA was extracted from human CRC tissue sections, and quantitative reverse transcription polymerase chain reaction (qRT–PCR) was performed as previously described.^9^ For qRT–PCR, the TaqMan gene expression assay for human *ITGB6* was used (Assay ID: Hs00168458_m1, catalogue 4331182, Thermo Fisher Scientific).^21^ The padded amplicon detected by the kit was as follows: (5′‐3′) TGGCGGAATGACTCCCTCCACCTCCTGGTCTTTGTGAGTGATGCTGATTCTCATTTTGGAATGGACAGCAAACTAGCAGGCATCGTCATTCCTAATGACGGGCTCTGTCACTTGGACAGCAAGAATGAATACTCCATGTCAACTGTCTTGGAATATCCAACAATTGGACAACTCATTGATAAACTGGTACAAAACAACGTGTTATTGATCTTCGCTGTAACCCAAGAACAAGTTCATTTATATGAGAATTACGCAAAACTTATTCCTGGAGCTACAGTAGGTCTACTTCAGAAGGACTCCG.

### 2.13 Statistical analysis

In the case of normally distributed data, 2‐tailed, unpaired or paired Student's *t* tests were used for statistically analyzing pairwise comparisons. For non‐normally distributed data, a 2‐tailed Mann–Whitney *U* test was used. GraphPad Prism software version 9.00 (GraphPad Software) was used for all the analyses. *p* values less than 0.05 were considered significant. Bar diagrams with mean ± standard deviation (SD) are shown.

## 3 RESULTS

### 3.1 *ITGB6* ‐expressing tumour cells bind to fibronectin‐expressing endothelial cells

Integrin αvβ6 binds to fibronectin.^7^ As the limiting binding partner of integrin αvβ6, integrin β6 plays a central role in controlling the expression and function of the heterodimer. FN is highly expressed by EC of the lung and liver, which are the primary sites of CRC metastasis, as well as in HUVEC (Figure 1A). As HUVECs show similar FN expression but are easier to handle, cheaper in maintenance, and faster in proliferation, they were used in the following experiments as a model system. The CRC cell lines HT‐29 and DLD‐1 did not express FN (Figure 1A). To study the potential interaction of integrin αvβ6 with fibronectin between tumour cells and EC, *ITGB6* expression in different CRC cell lines was investigated via western blotting. *ITGB6* was highly expressed in SW948 and HT‐29 cells but was not expressed in LOVO and RKO cells (Figure 1B).

Integrins are critical mediators of cell adhesion. IL‐1 enhances endothelial adhesiveness in cancer cells.^22^, ^23^, ^24^, ^25^ Accordingly, the intrinsic adhesive capacity of different CRC cell lines to IL‐1β‐stimulated HUVEC was investigated via a static adhesion assay. Tumour cells with high *ITGB6* expression (SW948 and HT‐29 cells) exhibited strong adhesion, whereas *ITGB6* nonexpressing cells (LOVO and RKO cells) showed significantly reduced adhesion (Figure 1C,D). These findings indicate the potential involvement of integrin αvβ6 in the adhesion of CRC tumour cells to EC.

### 3.2 *ITGB6* knockout reduces cancer cell adhesion to endothelial cells under static and flow conditions

To further explore the functional role of integrin αvβ6 in the adhesion of tumour cells to EC, we employed CRISPR/Cas9 technology to establish an *ITGB6* knockout (KO) cell line, based on the method outlined previously.^26^ HT‐29 cells were selected for this experiment due to their robust proliferation rate and naturally high levels of *ITGB6* expression. To achieve this, we utilized a single‐guide RNA (sgRNA) specifically targeting *ITGB6*, alongside a non‐targeting (NT) control sgRNA. Following transfection, KO and control (NT) cells were generated. These cells underwent single‐cell selection and were expanded into clonal populations. From these, two distinct clones were chosen for detailed analysis.

The knockout of *ITGB6* was confirmed through two complementary methods: western blotting (Figure 1E) and immunocytochemical staining (Figure 1F), both of which verified the absence of integrin β6 in the KO clones. Moreover, the functional consequences of *ITGB6* knockout were investigated. Staining for cleaved caspase‐3 and PARP showed a heightened apoptotic response in the *ITGB6* KO cells upon treatment with Nutlin‐3, a known apoptosis inducer (Figure 1G,H). This increase in apoptosis highlights the involvement of integrin β6 in survival pathways, with the KO cells exhibiting greater sensitivity to apoptosis compared to the control cells. Furthermore, wound healing assays revealed a marked decrease in the migratory capacity of *ITGB6* KO cells (Figure 1I,J), aligning with previously published results.^11^, ^12^, ^15^ The consistency of these observations with earlier findings strongly reinforces the successful knockout of *ITGB6* and its functional consequences in this model.

Additionally, we examined the effects of TNF‐α, a well‐established inducer of *ITGB6* expression,^27^ on NT cells. Treatment with TNF‐α for either 24 or 72 h resulted in a pronounced increase in *ITGB6* expression in control (NT) cells, as observed by western blotting (Figure 1K). Consistent with this, TNF‐α‐stimulated NT tumour cells displayed a significant enhancement in adhesion to HUVEC (Figure 1L). However, in *ITGB6* KO cells, no increase in adhesion was observed following TNF‐α stimulation (Figure 1L), demonstrating the specificity of integrin αvβ6 in mediating this adhesive interaction. Notably, tumour cells were washed after TNF‐α treatment to ensure that TNF‐α did not act on endothelial cells during the adhesion assay. Complete integrity of the HUVEC monolayer was controlled for this and every following experiment by phase contrast microscopy and is shown exemplarily (Figure 1L, insets; enlarged images Figure S1).

To further investigate the role of integrin αvβ6 in tumour cell adhesion under more physiologically relevant conditions, we employed flow adhesion assays. These assays were conducted under low shear stress (2.03 dyn/cm^2^), mimicking the conditions of capillary blood flow where tumour cells predominantly adhere.^28^ In these assays, *ITGB6* KO cells exhibited significantly reduced adhesion to HUVEC compared to control cells, further underscoring the critical role of integrin αvβ6 in promoting cell adhesion under flow conditions (Figure 1M). This decreased adhesion, even under conditions that closely resemble those within the microvasculature, strongly emphasizes the importance of integrin αvβ6 in facilitating the interaction of CRC tumour cells with EC.

To explore potential compensatory mechanisms for the loss of integrin β6, we examined whether other integrins, particularly integrin αv, were affected by integrin β6 deficiency. Integrin αv is known to partner with several β integrins, including integrin β6. Therefore, coimmunoprecipitation experiments were performed to assess the interaction of integrin αv with other integrins, such as integrin β5 and integrin β1, in both *ITGB6* KO and control (NT) cells. Lysates from HT‐29 KO and NT cells were immunoprecipitated with an anti‐integrin αv antibody, resolved by SDS–PAGE, and probed with antibodies specific for integrin β6, integrin β5, and integrin β1 (Figure S2). The results showed no significant difference in integrin β5 binding with integrin αv between the *ITGB6*‐expressing control cells and the KO cells. However, an increased interaction between integrin αV and the mature integrin β1 transcript was detected in the *ITGB6* KO cells, likely reflecting a compensatory mechanism due to the absence of integrin β6 (Figure S2).

Despite this increased integrin αvβ1 interaction, it was insufficient to restore the adhesive function of the *ITGB6* KO cells. The adhesion of *ITGB6* KO cells to HUVEC remained significantly impaired, even with the altered integrin αvβ1 interaction (Figure 1L,M). These findings suggest that integrin β6 plays a unique and non‐redundant role in mediating tumour cell adhesion to EC and that the compensatory interaction of integrin αv with integrin β1 is unable to fully substitute for integrin β6 in this process. This highlights the specific importance of integrin αvβ6 in promoting the adhesion of CRC cells to EC and supports its potential role in facilitating metastasis.

### 3.3 *ITGB6* on CRC tumour cells binds to fibronectin

**FIGURE 2:**
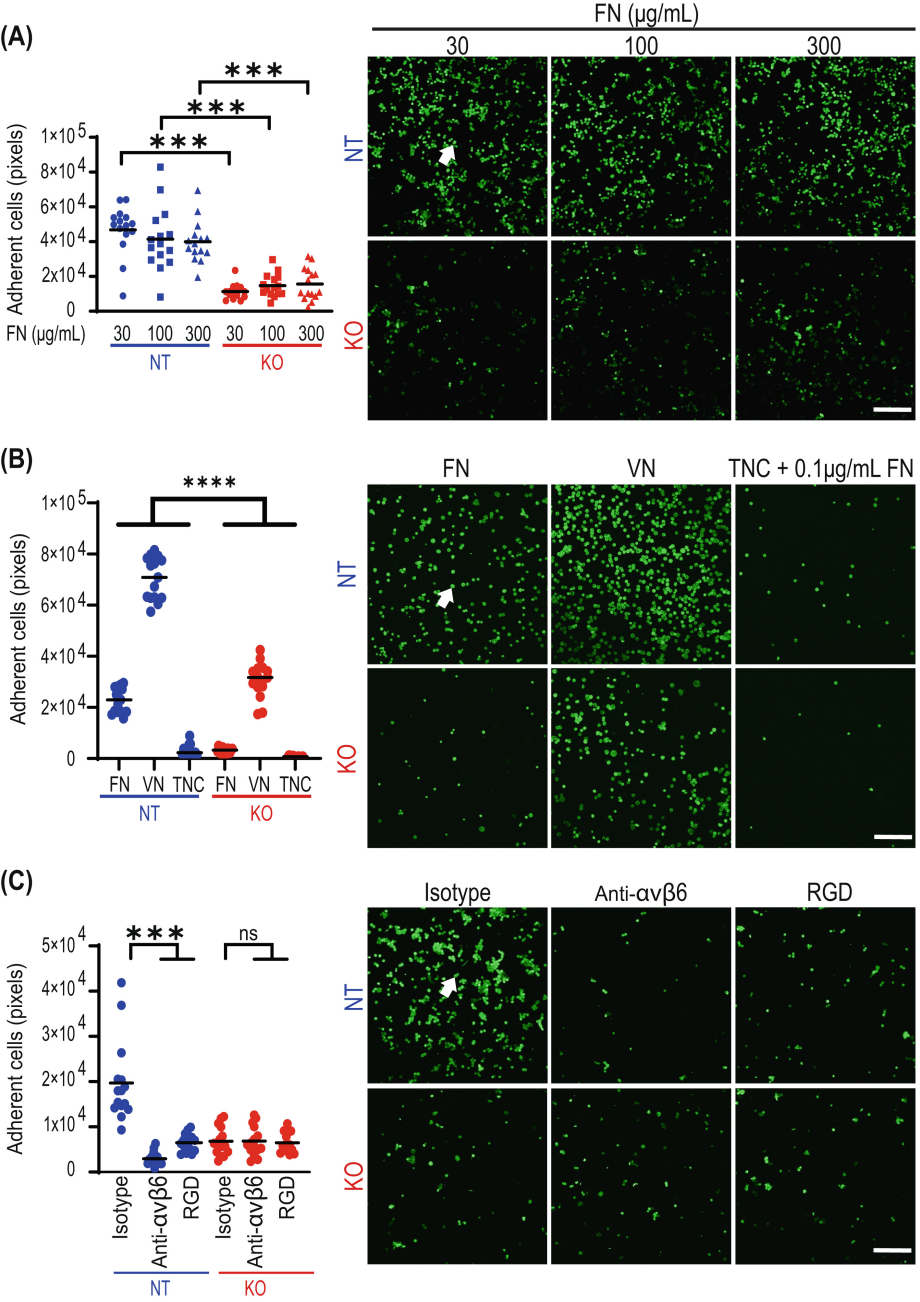
Integrin β6 is required for HT‐29 cell adhesion to fibronectin. (A) HT‐29 cells were plated on surfaces coated with the indicated fibronectin concentrations (μg/ml) for 4 h. Scale bar: 250 μm. Mann–Whitney *U* test; ***p < 0.001. (B) HT‐29 cells were plated on surfaces coated with 30 μg/mL fibronectin, 30 μg/mL vitronectin or 30 μg/mL tenascin C plus 0.1 μg/mL fibronectin for 4 h. Scale bar: 250 μm. Mann–Whitney *U* test; ****p < 0.0001. (C) HT‐29 cells were pretreated for 10 min with an isotype control (anti‐mouse IgG2a), anti‐αvβ6 antibody or RGD peptide before being plated on surfaces coated with 30 μg/mL fibronectin for 4 h in the presence of the treatment. Scale bar: 250 μm. Mann–Whitney *U* test; ns, not significant; ***p < 0.001. CRC cells were stained using Cytopainterr. Adherent cells are marked by arrows.

To investigate the binding of integrin αvβ6 on tumour cells to fibronectin, an adhesion assay on fibronectin‐coated surfaces was performed. The adhesion of *ITGB6*‐expressing NT cells was significantly increased at all concentrations of coated fibronectin (Figure 2A). Adhesion assays on alternative binding substrates of integrin β6 showed that αvβ6‐expressing NT cells adhered strongly to vitronectin and not or only weakly to tenascin C (Figure 2B). Of note, tumour cells showed a very high binding activity to vitronectin also in the absence of β6 expression (Figure 2B). These results indicated that β6 on tumour cells mediates predominantly binding to fibronectin, adhesion to vitronectin also involves other molecules, and tenascin C binds only very weakly to β6.

The minimal recognition sequence on extracellular matrix proteins such as fibronectin, vitronectin, and tenascin C required for binding of αvβ6 integrin is an RGD (Arg‐Gly‐Asp) motif. Accordingly, the increased adhesion of *ITGB6*‐expressing NT cells on fibronectin could be reduced by blocking the minimal recognition site for integrin binding with an RGD peptide and, more specifically, blocking integrin β6 with a neutralizing anti‐integrin αvβ6 antibody, demonstrating the specificity of the interaction (Figure 2C).

**FIGURE 3:**
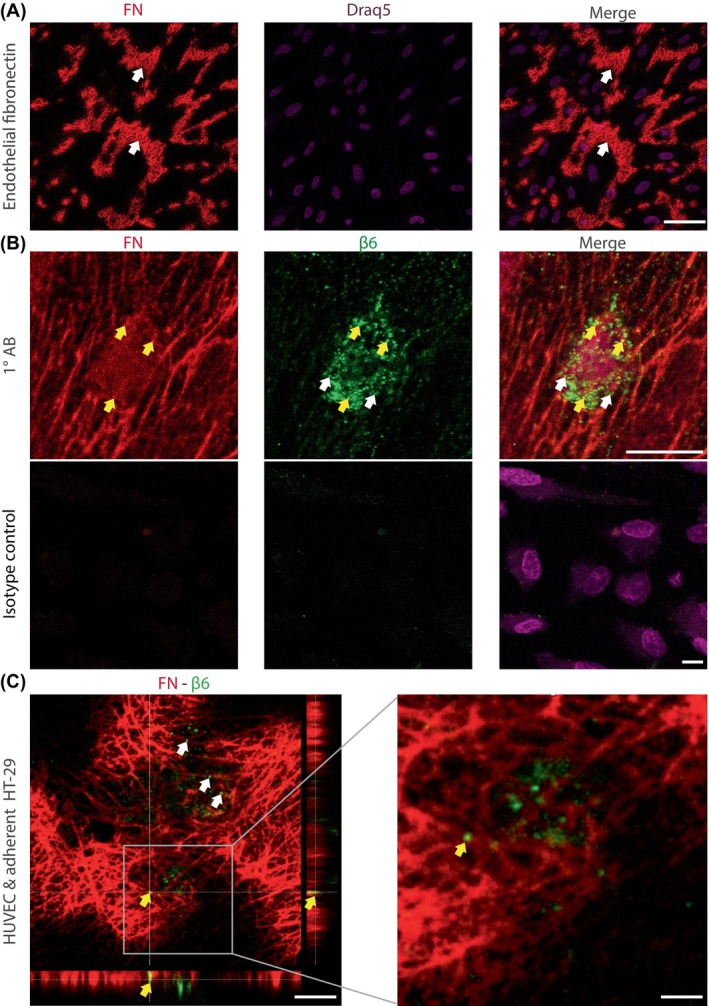
Endothelial fibronectin and integrin β6 from tumour cells colocalize during adhesion. (A) HUVEC were fixed and subjected to immunofluorescent staining for fibronectin (red, arrows) and Draq5 (purple). Scale bar: 50 μm. (B) Tumour cells adherent to HUVEC were fixed and subjected to immunofluorescent staining for fibronectin (red), integrin β6 (green, white arrows) and Draq5 (purple). Colocalization of integrin β6 and fibronectin is indicated by yellow arrows. Scale bar: 10 μm. (C) Orthogonal sectioning of the HUVEC and adherent tumour cells confirms the colocalization (yellow arrows) of fibronectin (red) and integrin β6 (green, white arrows). Scale bar: 10 μm; enlarged image: 2 μm.

Fibronectin depositions by HUVEC were shown by immunofluorescent staining (Figure 3A, arrows). Co‐staining of endothelial fibronectin deposits and of β6 on tumour cells indicated close contact of both molecules (Figure 3B, yellow arrows), which was further confirmed by overlapping staining patterns in the optical Z‐plane (Figure 3C, yellow arrow, Video S1). A stepwise focusing through the three‐dimensional complex of an *ITGB6*‐expressing tumour cell (Video S2, green) attached to a fibronectin‐expressing endothelial cell (Video S2, red) confirmed the interaction of both molecules (Video S2, yellow arrows).

### 3.4 Neutralization of integrin αvβ6 on tumour cells reduces adhesion to endothelial cells

**FIGURE 4:**
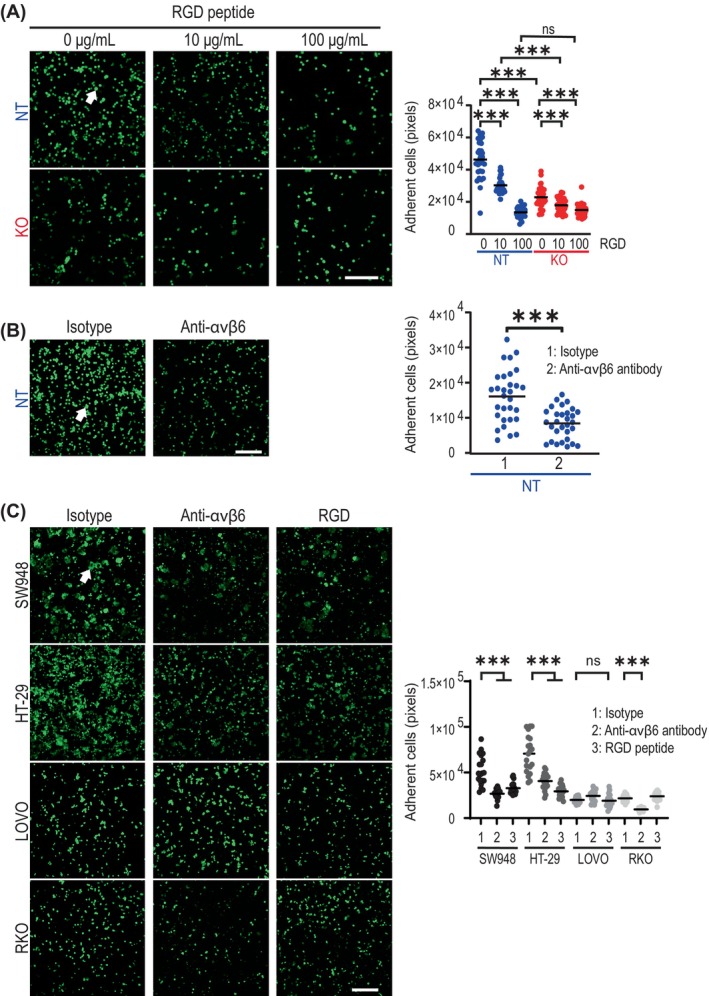
Blocking integrin β6 reduces cancer cell adhesion to endothelial cells. (A) Adhesion of HT‐29 cells to an HUVEC monolayer in the presence of the RGD peptide at the indicated concentrations after 2 h of incubation. Scale bar: 250 μm. Mann–Whitney *U* test; ns, not significant; ***p < 0.001. (B) Adhesion of pretreated HT‐29 cells to a HUVEC monolayer in the presence of 10 μg/mL anti‐αvβ6 antibody after 2 h of incubation. Scale bar: 250 μm. Mann–Whitney *U* test; ***p < 0.001. (C) Adhesion of *ITGB6*‐expressing CRC cells (SW948, HT‐29) on an HUVEC monolayer can be blocked by 10 μg/mL RGD peptide and 10 μg/mL anti‐αvβ6 antibody. CRC cell lines with low *ITGB6* expression (LOVO, RKO) exhibit low adhesion to HUVEC. Scale bar: 250 μm. Mann–Whitney *U* test; ns, not significant; ***p < 0.001. In A, B, and C, IL‐1β‐stimulated HUVEC were used, and CRC cells were stained using cytopainter. Adherent cells are indicated by arrows.

Next, we tested whether the RGD peptide (Figure 4A) and the anti‐integrin αvβ6 antibody (Figure 4B) inhibit tumour cell binding to EC. A dose‐dependent effect of blocking was observed for the RGD peptide (Figure 4A). In addition, significant inhibition was observed with the anti‐integrin αvβ6 antibody (Figure 4B). The lower adhesion of *ITGB6*‐expressing cells (HT‐29, SW948) in the presence of the neutralizing anti‐integrin αvβ6 antibody and the RGD peptide, as well as the low adhesion of naturally low‐*ITGB6*‐expressing cells (LOVO, RKO), was confirmed in additional cell lines (Figure 4C). Overall, these results support the direct involvement of integrin β6 in the adhesion of tumour cells to EC.

### 3.5 Integrin β6 mediates the proteolysis‐resistant adhesion of CRC cells to endothelial cells

**FIGURE 5:**
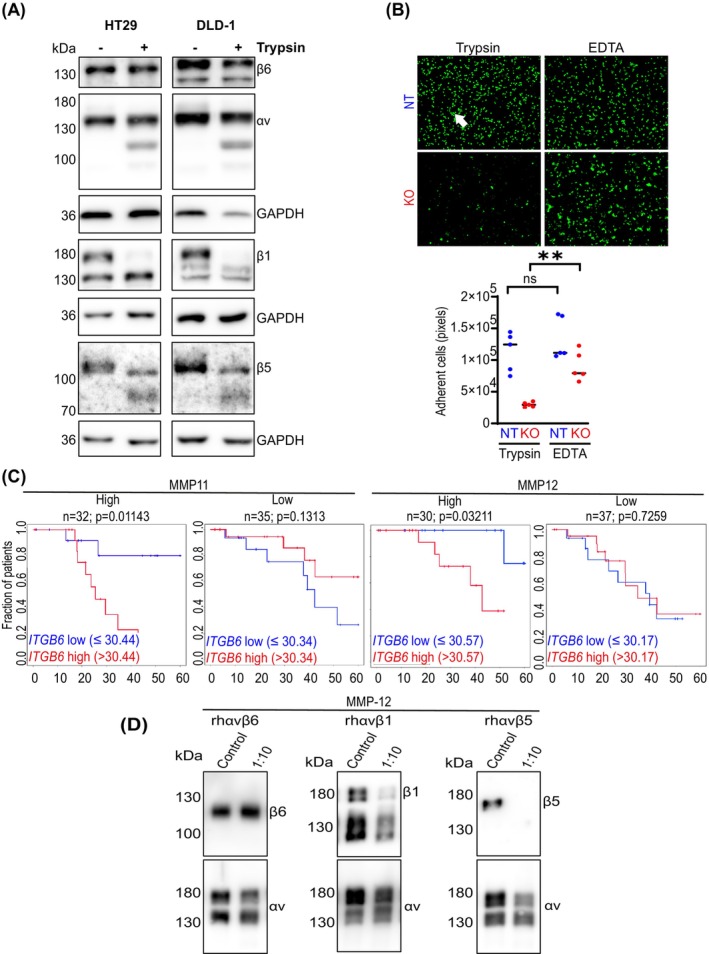
Integrin β6 is a proteolysis‐resistant mediator of CRC tumour cell adhesion to endothelial cells. (A) Lysates of HT‐29 and DLD‐1 cells harvested in the presence of 0.05% trypsin or 5 mM EDTA‐PBS were collected, and the expression of integrins αv, β6, β1 and β5 was analysed using western blotting. Blots show the full‐length proteins as well as the trypsin‐induced protein fragments. GAPDH was used as a loading control. (B) Adhesion of cytopainter‐stained *ITGB6* KO or NT cells harvested by trypsinization or 5 mM PBS‐EDTA to an IL‐1β‐stimulated HUVEC monolayer under static conditions. Arrow points towards an adherent cell. Scale bar: 250 μm. The quantification is shown below. Mann–Whitney *U* test; ns, not significant; **p < 0.01. (C) The cancer‐related survival (60‐month cut‐off) of CRC patients who did not receive neoadjuvant therapy and had no residual tumour (R0) was explored. Based on MMP11 and MMP12 RNA expression levels, patients were divided into high‐ and low‐MMP groups. *ITGB6*‐dependent survival was investigated in these groups. The number of patients per group is indicated by ‘*n*’. Cox proportional hazards survival regression. (D) Recombinant integrins αvβ6, αvβ1, and αvβ5 were incubated with MMP‐12 at an enzyme‐to‐substrate ratio of 1:10 for 1 h. Proteolytic cleavage of integrins was evaluated via western blotting.

Metastatic tumours often exhibit high proteolytic activity, which might affect the integrity of proteins on the cell surface.^29^, ^30^, ^31^ To investigate the impact of externally applied proteolytic activity on integrin structure, both the poorly invasive HT‐29 cells and the highly invasive DLD‐1 cells^32^ were subjected to trypsin digestion, and surface integrins were analysed via western blotting (Figure 5A). Interestingly, with the exception of integrin β6, all integrin αv‐interacting integrins expressed in both CRC cell lines were cleaved by this approach (Figure 5A). Given that both cell lines express a different panel of MMPs,^33^ exhibit differential invasiveness,^32^ different microsatellite stability (HT‐29: MSS; DLD‐1: MSI)^34^ and CMS status (HT‐29: CMS3, DLD‐1: CMS1)^35^ proteolytic stability of integrin β6 seems to be a characteristic feature of CRC tumour cells. Next, the adhesion of *ITGB6*‐expressing NT and nonexpressing KO cells to EC after trypsin digestion was investigated. Trypsin treatment did not decrease the adhesion of NT control cells (Figure 5B). In contrast, in KO cells, the decrease in adhesion was significantly further reduced by trypsin (Figure 5B). These findings indicate that trypsin‐resistant integrin β6 is the primary mediator of CRC cell adhesion. Residual lower adhesion in the absence of integrin β6 is mediated by trypsin‐sensitive integrins.

Under consideration, that integrin β6 may be a proteolysis‐resistant supporter of metastasis, its impact on patient metastasis and survival should be predominantly noticeable in patients with highly proteolytically active tumours. Proteolysis during tumour‐associated tissue remodelling is mediated by MMPs. Interestingly, we found that *ITGB6* expression was associated with significantly reduced cancer‐related survival in tumours with high MMP‐11 and MMP‐12 expression but not in those with low expression (Figure 5C, patient characteristics: Table S1). The differential expression of MMP‐7 and MMP‐9 did not affect the impact of *ITGB6* on survival (Figure S3).

In order to further investigate the possible resistance of β6 to proteolytic cleavage, we analyzed the sensitivity of the integrins β1, β5, β6 and αv to MMP‐12. MMP‐12 was selected because its expression is increased in CRC,^36^ in patients with high MMP‐12 expression prognosis is associated with ITGB6 expression (Figure 5C), and MMP‐12 is commercially available. Remarkably, we found that MMP‐12 cleaved the recombinant integrins β1 and β5, whereas αv and again β6 were resistant (Figure 5D). This supports the hypothesis that integrin β6 is a proteolysis‐resistant mediator of adhesion between tumour cells and EC, which contributes to metastasis and worsened patient survival.

### 3.6 *ITGB6* ‐expressing tumour cells are enriched in liver metastases of CRC

**FIGURE 6:**
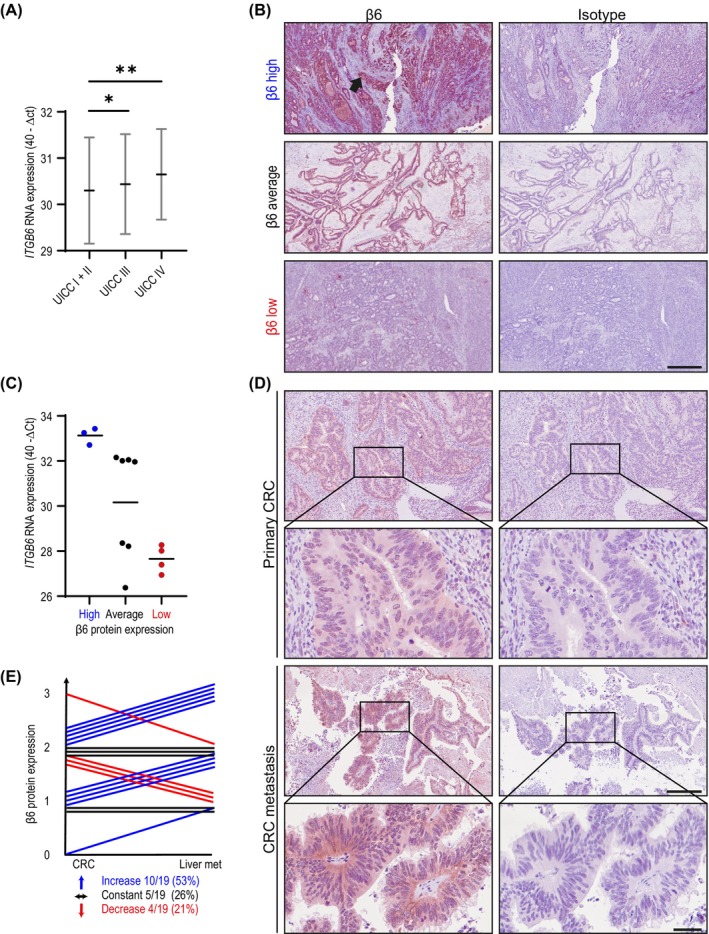
*ITGB6* expression is correlated with tumour stage and highly increased in liver metastases of colorectal cancer. (A) The association of *ITGB6* expression with the UICC 2017 stages of 464 CRC patients. Mann–Whitney *U* test; *p < 0.05; **p < 0.01. (B) Different grades of IHC staining for integrin β6 in primary CRC tissues. Staining signals are indicated by an arrow. Scale bar: 500 μm. (C) Comparison of *ITGB6* expression measured in CRC tissues (*n* = 14) using qRT–PCR and IHC. (D) IHC staining for integrin β6 in primary CRC tissue and liver metastatic lesions from the same patients (*n* = 19). Scale bar: 250 μm; enlarged image: 50 μm. (E) Comparison of integrin β6 expression in primary CRC tissues and liver metastases from the same patients. 0, negative; 1, low expression; 2, medium expression; 3, high expression. Increased expression in the liver metastatic tissue compared with the CRC tissue is indicated in blue. No change in expression is noted in black. Decreased expression is noted in red.

*ITGB6* expression was increased in the progressive stages of CRC, as shown by qRT–PCR results for 464 patients (Figure 6A, patient characteristics: Table S2). In order to validate the specificity of the immunohistochemical integrin β6 staining results, we investigated whether *ITGB6* RNA expression is related to protein expression. To this goal, *ITGB6* RNA and protein expression were analysed in 14 patients (Figure 6B,C, patient characteristics: Table S3). The results revealed that high and low *ITGB6* RNA expression were closely related to the strong and low signals obtained with the anti‐integrin β6 antibody via immunohistochemical staining, respectively (Figure 6B,C). These findings indicate that the integrin β6 protein can be specifically detected in CRC tissues via immunohistochemistry. Using the specific detection method, integrin β6 protein expression was analysed in a further cohort of 19 patients, both in primary CRC and the corresponding liver metastases of the same patients (patient characteristics: Table S4). Integrin β6 expression was higher in the tumour cells in the metastases than in those in the primary lesions (Figure 6D) in 10 of the 19 patients (53%, Figure 6E). In 5 of the remaining patients, integrin β6 expression was not altered (26%), and a decrease (21%) was observed in only 4 patients (Figure 6E). These results suggest that integrin β6‐expressing cells are enriched at the metastatic site.

## 4 DISCUSSION

Our study revealed that the integrin β6 subunit, as the limiting partner of the integrin αvβ6 heterodimer, has the potential to assist in the formation of metastases in CRC patients. Hereby, it corroborates previous findings of a correlation between *ITGB6* expression and poor patient survival.^8^, ^14^ Our findings that an increased number of patients exhibit high *ITGB6* expression in metastatic lesions in the liver compared with primary CRC tissue support a role of integrin β6 in metastatic colonization, highlighting its clinical relevance. Integrins are critical mediators of cell adhesion and migration and have been implicated in cancer progression.^37^ Integrin αvβ6 is associated with CRC pathogenesis and has emerged as a potential mediator in the adhesion of disseminated tumour cells to EC, a critical step in the metastatic cascade.

The attachment of cancer cells to the endothelium involves integrins and their ligands.^4^ Integrin–ligand interactions can be divided into four distinct classes, one of which is the RGD‐binding integrins, including integrin αvβ6.^38^ This class of integrins is characterized by their tripeptide RGD (Arg‐Gly‐Asp) recognition motif formed in the interface of the α and ß subunits, through which they interact collaboratively with RGD‐containing extracellular matrix proteins. Previously, *ITGAVB3* expressed by tumour cells was reported to bind to L1‐CAM found on EC.^4^ Although *ITGAVB3* is expressed on various types of tumour cells, the HT‐29 CRC tumour cells used in the present study were shown to be immunonegative for *ITGAVB3* (Figure S4). Therefore, their adhesion to EC may be attributed to a different mechanism.

One intriguing observation from our study was the resistance of integrin β6 to trypsinization, a phenomenon not observed for other integrins, such as integrin αv, integrin β1 and integrin β5. Notably, integrin β6 also showed lower sensitivity to cleavage by the tumour‐associated matrix metalloproteinase MMP‐12, in contrast to integrins β1 and β5, supporting its resilience to proteolysis in tumour‐relevant conditions. Similar reports showing the differential resistance of integrin subunits to proteases have been published by other researchers. M. A. Brown et al. reported the sensitivity of integrin α5β1 to trypsin but not that of integrin αvβ3.^39^ D. C. von Bredow et al. reported the cleavage of integrin β4 but not of integrin β1 or integrin α6 by matrilysin, also known as MMP‐7.^40^ Given that malignant tumours show high proteolytic activity, which is required for invasive growth^31^, ^41^ and for the induction of the angiogenic switch,^42^ resistance to proteases might impact metastasis development.

Our study suggests an interaction between integrin αvβ6 on tumour cells and fibronectin on EC. Previous work by Barbazán et al. showing the presence of fibronectin in the form of deposits on the luminal side of liver blood vessels in human livers affected by metastasis strongly supports our findings.^43^ Fibronectin is a specific ligand for integrin αvβ6. The present study revealed increased adhesion of *ITGB6*‐expressing cells on fibronectin‐coated surfaces, and this adhesion can be blocked by an RGD peptide and an anti‐integrin αvβ6 antibody. Together, these findings suggest that fibronectin deposits on EC in the target organs of CRC metastasis could be potential binding sites for tumour cells expressing integrin β6. Further experiments, such as in vivo studies with integrin β6‐blocking antibodies, are necessary to confirm this interaction's contribution to metastasis formation.

The ability of an RGD peptide or an anti‐integrin αvβ6 antibody to block the adhesion of tumour cells expressing *ITGB6* indicates that this interaction can be targeted. Previous studies have shown the effectiveness of integrin β6‐targeted therapy in reducing tumour growth.^7^ A novel, recombinant B6.3 diabody, developed to bind specifically to integrin αvβ6 and to target *ITGAVB6*‐expressing tumours, holds great potential. This ligand‐mimicking diabody elicits integrin αvβ6 internalization upon binding and blocks integrin αvβ6‐dependent adhesion and migration to fibronectin and LAP.^7^ These studies and the findings reported here strongly support that integrin αvβ6 is a promising anticancer therapeutic target for clinical practice.

In conclusion, our study elucidates a novel mechanism contributing to CRC metastasis, emphasizing the role of integrin β6 in mediating tumour cell adhesion to EC, particularly through its interaction with fibronectin deposits in liver blood vessels. These findings increase the knowledge of molecules involved in the very early contact of CRC cells with target tissues and provide new perspectives for the use of integrin β6 as a target in individualized therapeutic efforts against metastasis in CRC patients.

## AUTHOR CONTRIBUTIONS

**Chiara Van Passen:** Conceptualization; data curation; formal analysis; investigation; methodology; validation; visualization; writing – original draft. **Julia Krug:** Formal analysis; methodology. **Luisa Weiß:** Data curation; formal analysis; investigation; methodology. **Mariam Mohamed Abdou:** Methodology. **Philipp Tripal:** Methodology; investigation; resources. **Benjamin Schmid:** Formal analysis; visualization. **René Krüger:** Formal analysis. **Yanmin Lyu:** Formal analysis. **Bisan Abdalfatah Zohud:** Formal analysis; investigation. **Katja Petter:** Investigation. **Carol Geppert:** Resources. **Susanne Merkel:** Resources. **Barbara Bärthlein:** Resources. **Philipp Busenhart:** Supervision. **Michael Scharl:** Conceptualization; resources; funding acquisition; supervision. **Elisabeth Naschberger:** Funding acquisition; methodology; resources; supervision; writing – review and editing; visualization. **Michael Stürzl:** Conceptualization; data curation; formal analysis; funding acquisition; methodology; project administration; resources; supervision; validation; visualization; writing – review and editing.

## FUNDING INFORMATION

This work was supported by grants from the German Research Foundation (DFG) by the FOR 2438, subproject 2 (project ID 280163318) to E. Naschberger and M. Stürzl; by the DFG SFB/TRR 241, subproject A06 (project ID 375876048) to M. Stürzl; DFG STU 238/10‐1 (project ID 437201724) to M. Stürzl and M. Scharl; by the DFG TRR 305, subproject B08 (project ID 429280966) to E. Naschberger; by the DFG‐NOTICE program (project ID 493624887) to E. Naschberger; by the W. Lutz Stiftung to M. Stürzl; and by the Forschungsstiftung Medizin am Universitätsklinikum Erlangen to M. Stürzl. The Leica SP5 laser scanning microscope was funded by the DFG (project ID 52732026). Y. Lyu was sponsored by a Chinese Government Scholarship from the China Scholarship Council. C. Van Passen was supported by the Bavarian Equal Opportunities Sponsorship – Realisierung von Chancengleichheit von Frauen in Forschung und Lehre (FFL) – Realization of Equal Opportunities for Women in Research and Teaching.

## CONFLICT OF INTEREST STATEMENT

The authors declare that no conflicts of interest exist.

## ETHICS STATEMENT

Studies including patients were approved by the ethics committee (institutional review board) of the University Hospital of Erlangen (approval number: 3914, Polyprobe study). All participants were informed personally and provided written informed consent for this study. Patient data were pseudonymized, and all analyses were performed in accordance with the Helsinki declaration.

## Supporting information


**Data S1.** Supporting Information.

Video S1.

Video S2.

## Data Availability

All the results are reported in the manuscript. The original code designed and used in the current study to quantify the number of adherent cells is publicly available on GitHub (https://github.com/ChiaraVP/Macro-for-quantifying-adherent-cells.git). Further information is available from the corresponding author upon request.
