# Supplementary material for: Integrin β6 expression in colorectal cancer cells promotes liver metastasis through enhanced adhesion to endothelial fibronectin
Source: Int J Cancer. 2025 Jun 9;157(7):1481–95. doi: 10.1002/ijc.35504 (PMC12334909; doi:10.1002/ijc.35504)
Supplement: Supplementary file 1 — Data S1. Supporting Information. [file IJC-157-1481-s001.pdf]

Supplemental material

Integrin β6 expression in colorectal cancer cells promotes liver metastasis through enhanced adhesion to endothelial fibronectin

Chiara Van Passen, Julia Krug, Luisa Weiß, Mariam Mohamed Abdou, Philipp Tripal, Benjamin Schmid, René Krüger, Yanmin Lyu, Bisan Abdalfatah Zohud, Katja Petter, Carol Geppert, Susanne Merkel, Barbara Bärthlein, Philipp Busenhart, Michael Scharl, Elisabeth Naschberger, Michael Stürzl

Table of contents

Supplemental table 1. Clinical characteristics of the colorectal carcinoma patients included in the analysis of the patient survival 2

Supplemental table 2. Clinical characteristics of the colorectal carcinoma patients included in the analysis of the expression of ITGB6 in relation to the CRC stage distribution 3

Supplemental table 3. Clinical characteristics of the colorectal carcinoma patients included in the quantitative comparison of ITGB6 RNA and protein expression 4

Supplemental table 4. Clinical characteristics of the colorectal carcinoma patients included in the analysis of the comparison of β6 expression in primary and metastatic CRC lesions 5

Supplemental figure 1. Integrity of the HUVEC monolayer during tumour cell adhesion assays 6

Supplemental figure 2. Co-immunoprecipitation of integrin αv and its ligands in ITGB6 KO cells reveals no altered binding of integrin β5, but increased binding of integrin β1 in the absence of competition from integrin β6 7

Supplemental figure 3. No effect of ITGB6 on cancer-related survival in MMP7 and MMP9 high expressing patients 8

Supplemental figure 4. Lack of αvβ6 expression by HT-29 cells 9

Supplemental video 1. Uploaded separately
Supplemental video 2. Uploaded separately

Supplemental table 1. Clinical characteristics of the colorectal carcinoma patients included in the analysis of the patient survival (Fig. 5C and S3).

**Characteristics**	**n = 464**	**%**
**Male:female ratio**	273:191 = 1.43	NA
**Mean/range age (years)**	67.8/ 20-96.9	NA
**Pathological stage (UICC 2017)**		
I	131	28.2
II	147	31.7
III	111	23.9
IV	75	16.2
**Histopathological grading**		
Low grade (G1/ G2)	318	68.5
High grade (G3/G4)	143	30.8
Missing information	3	0.6

NA = not applicable

Supplemental table 2. Clinical characteristics of the colorectal carcinoma patients included in the analysis of the expression of *ITGB6* in relation to the CRC stage distribution (Fig. 6A).

**Characteristics**	**n = 351**	**%**
**Male:female ratio**	205:146 = 1.40	NA
**Mean/range age (years)**	68.5/ 24-96	NA
**Pathological stage (UICC 2017)**		
I	76	21.7
II	121	34.5
III	89	25.4
IV	65	18.5
**Histopathological grading**		
Low grade (G1/ G2)	228	65.0
High grade (G3/G4)	120	34.2
Missing information	3	0.9

NA = not applicable

Supplemental table 3. Clinical characteristics of the colorectal carcinoma patients included in the quantitative comparison of *ITGB6* RNA and protein expression (Fig. 6B-C).

Pat	ITGB6 tissue	UICC Stage	Localisation	Neoadjuvant radio- or chemotherapy	Grade	T	N	M	L	V	R	Age	Sex
1	2	III	transverse colon	none	3	3	2	0	1	0	0	84	m
2	1	I	sigmoid colon	none	2	2	0	0	0	0	0	67	m
3	1	I	cecum	none	3	2	0	0	0	0	0	62	f
4	1	II	sigmoid colon	none	2	3	0	0	1	0	0	74	m
5	2	II	descending colon	none	2	3	0	0	0	0	0	52	f
6	1	IV	ascending colon	none	3	3	2	1	1	1	0	27	m
7	2	II	sigmoid colon	none	2	3	0	0	0	0	0	67	m
8	2	II	ascending colon	none	2	3	0	0	0	0	0	70	m
9	2	II	ascending colon	none	2	3	0	0	1	0	0	75	m
10	2	IV	sigmoid colon	none	3	4	2	1	0	0	2	82	f
11	2	III	ascending colon	none	2	4	2	0	1	0	0	60	f
12	3	II	cecum	none	2	4	0	1	1	0	0	77	m
13	3	II	sigmoid colon	none	3	3	0	0	0	0	0	57	m
14	3	IV	cecum	none	3	4	2	0	1	0	2	68	m

T = Size and extent of the primary tumour (T0: No primary tumour detectable, T1-4: Primary tumour of increasing size or depth of invasion); **N** = Number and location of affected lymph nodes (N0: No lymph node involvement detectable, N1-3: Increasing infestation of lymph nodes near the tumour), **M** = Occurrence of distant metastases (M0: No distant metastases detectable, M1: Detection of distant metastases at one or more sites); **L** = Spread of cancer cells in the lymph vessels (L0: No, L1: Yes); **V** = Spread of cancer cells in the blood vessels (V0: No, V1: Yes); **R** = Residual tumour (R0: Complete resection, R1: Microscopically positive resection margin, R2: Gross unresected tumour remaining, RX: Margin cannot be assessed).

Supplemental table 4. Clinical characteristics of the colorectal carcinoma patients included in the analysis of the comparison of β6 expression in primary and metastatic CRC lesions (Fig. 6D-E).

Pat	ITGB6 1° tissue	ITGB6 met tissue	UICC Stage	Localisation	Neoadjuvant radio- or chemotherapy	Grade	T	N	M	L	V	R	Age	Sex
1	1	2	IV	rectum	none	2	2	1	1	0	0	0	75	m
2	1	1	IV	sigma	none	3	4	2	1	1	0	0	60	m
3	2	1	IV	transverse colon	none	2	4	2	1	1	1	0	69	m
4	1	2	IV	sigma	none	2	3	2	1	1	0	X	52	m
5	2	2	IV	sigma	none	2	3	1	1	0	0	0	72	m
6	1	2	IV	descending colon	none	3	4	1	1	1	0	0	55	f
7	2	3	IV	ascending colon	none	3	3	1	1	1	0	0	67	m
8	0	1	IV	cecum	none	3	3	2	1	1	0	0	80	m
9	2	3	IV	flexura lienalis	none	2	3	2	1	1	0	X	53	f
10	2	1	IV	transverse colon	none	3	4	2	1	1	0	1	67	f
11	2	1	IV	sigma	none	2	3	1	1	0	0	0	70	m
12	2	3	IV	ascending colon	none	2	3	1	1	0	0	0	71	m
13	2	3	IV	flexura hepatica	none	3	4	0	1	0	1	X	71	m
14	3	2	IV	appendix	none	3	-	0	1	1	0	2	53	m
15	2	2	IV	rectum	none	3	3	0	1	0	0	0	57	m
16	2	2	IV	flexura lienalis	none	3	4	2	1	1	1	2	65	f
17	1	2	IV	ascending colon	none	2	2	0	1	0	1	0	66	f
18	2	3	IV	rectum	none	2	3	0	1	0	0	0	67	f
19	1	1	IV	cecum	none	3	2	1	1	1	0	1	77	m

T = Size and extent of the primary tumour (T0: No primary tumour detectable, T1-4: Primary tumour of increasing size or depth of invasion); **N** = Number and location of affected lymph nodes (N0: No lymph node involvement detectable, N1-3: Increasing infestation of lymph nodes near the tumour), **M** = Occurrence of distant metastases (M0: No distant metastases detectable, M1: Detection of distant metastases at one or more sites); **L** = Spread of cancer cells in the lymph vessels (L0: No, L1: Yes); **V** = Spread of cancer cells in the blood vessels (V0: No, V1: Yes); **R** = Residual tumour (R0: Complete resection, R1: Microscopically positive resection margin, R2: Gross unresected tumour remaining, RX: Margin cannot be assessed).

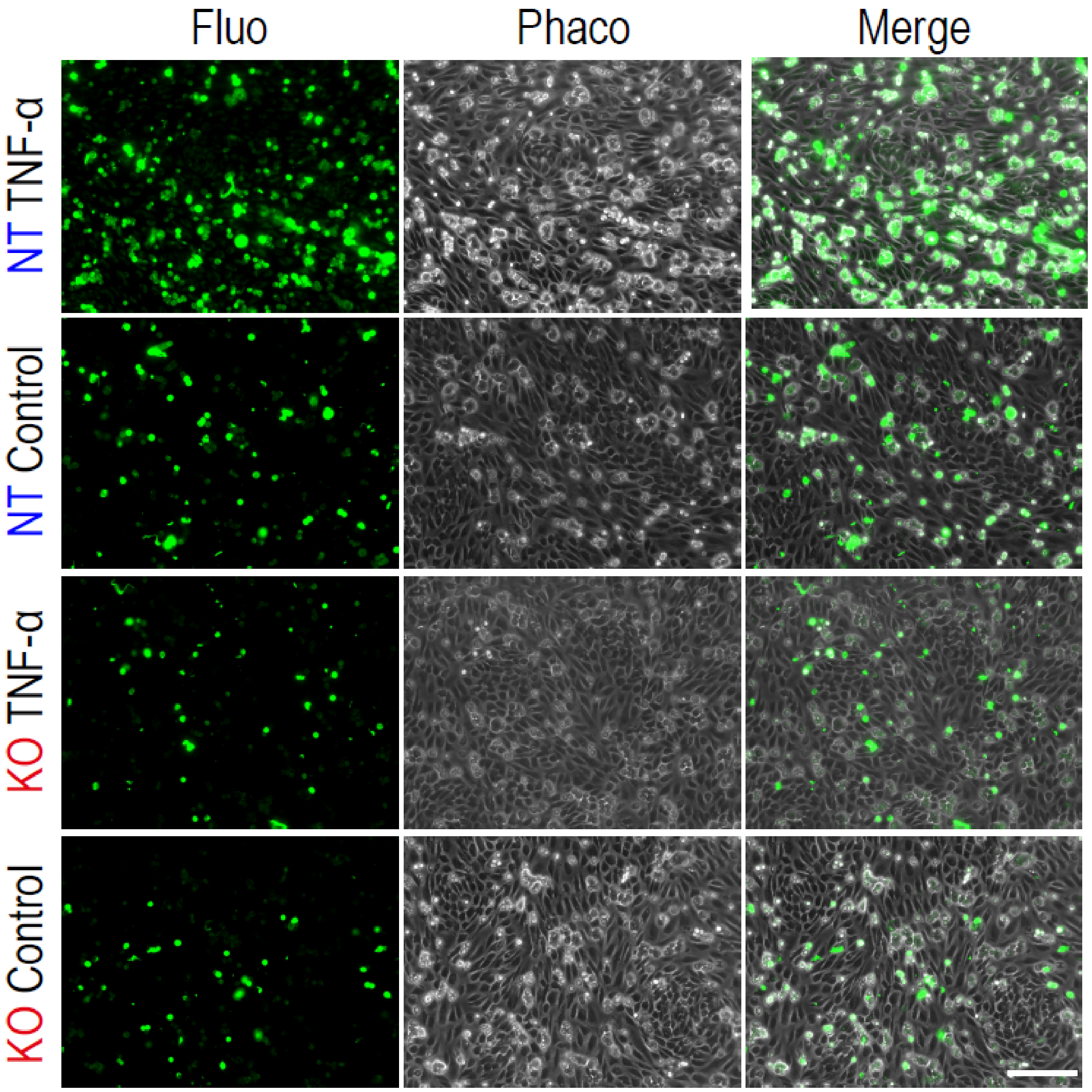

Supplemental figure 1. Integrity of the HUVEC monolayer during tumour cell adhesion assays. Representative fluorescence (Fluo), phase contrast (Phaco), and merged images demonstrating the integrity of the HUVEC monolayer under different experimental conditions. HT-29 NT and KO cells were allowed to adhere to the endothelial monolayer with or without TNF-α stimulation. Phase contrast images confirm that the HUVEC monolayer remains intact during the assay, ensuring that tumour cell adhesion occurs on endothelial cells rather than directly on the extracellular matrix. Scale bar, 250 µm.

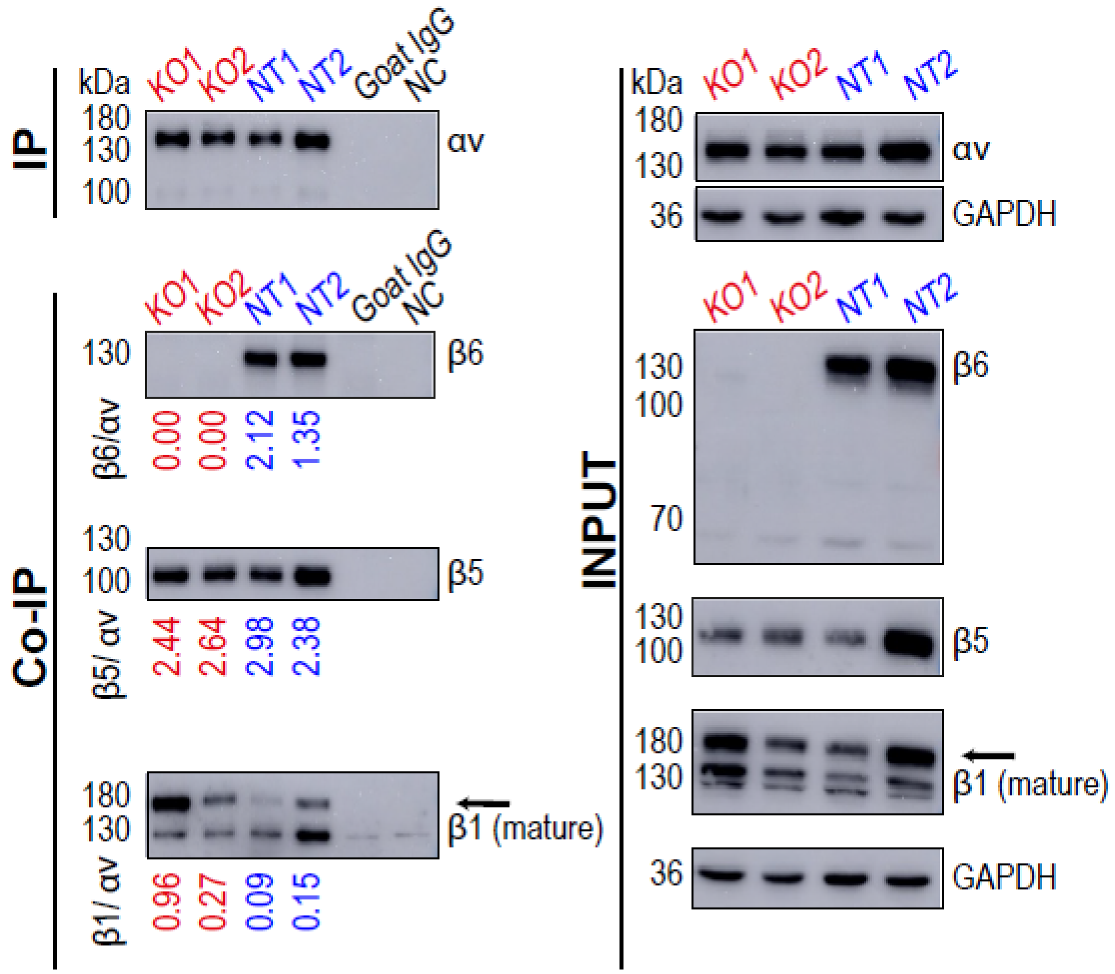

Supplemental figure 2. Co-immunoprecipitation of integrin αv and its ligands in *ITGB6* KO cells reveals no altered binding of integrin β5, but increased binding of integrin β1 in the absence of competition from integrin β6.

Lysates of HT-29 cells were collected, immunoprecipitated with protein G beads and anti-αv antibody. Immunoprecipitated lysates analysed with western blotting in the presence of anti-β6 integrin, anti-β5 integrin and anti-β1 integrin. GAPDH was used as loading control and goat-derived IgG isotype antibody (Goat IgG) and beads (NC) were used as controls.

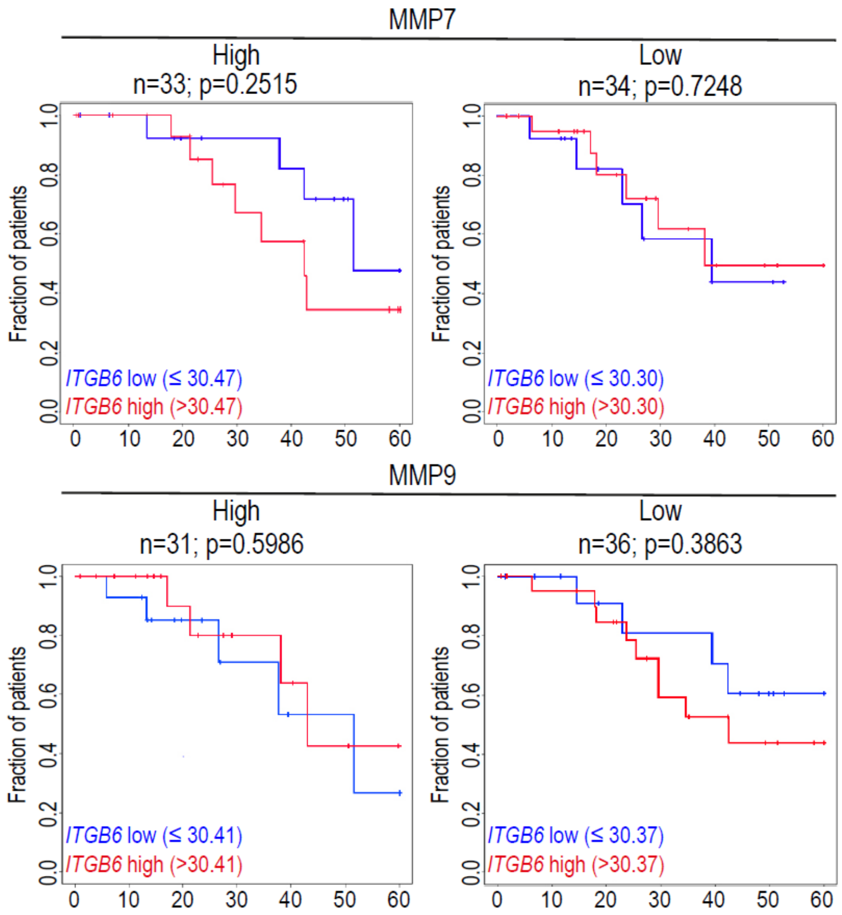

Supplemental figure 3. No effect of *ITGB6* on cancer-related survival in MMP7 and MMP9 high expressing patients.

Patients were divided in those with high and low MMP7 and MMP9 RNA expression. The cancer related survival (60 months cut-off) in relation to the *ITGB6* expression is shown. Patients did not receive neoadjuvant therapy and had no residual tumour (R0). The number of patients per group are indicated by 'n'. Cox Proportional Hazards Survival Regression.

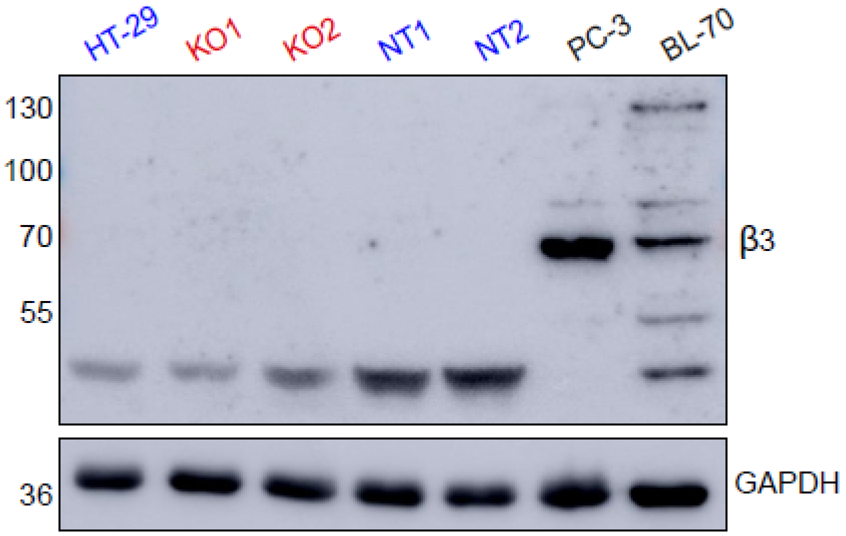

Supplemental figure 4. Lack of αvβ6 expression by HT-29 cells.

Using western blot analysis, HT-29 CRC tumour cells were shown to be negative for integrin αvβ3. PC-3 and BL-70 cells were used as positive controls for β3 expression.
